# Search graph structure and its implications for multi-graph constrained routing and scheduling problems

**DOI:** 10.1038/s41598-022-18026-w

**Published:** 2022-09-01

**Authors:** Michal Weiszer, Edmund K. Burke, Jun Chen

**Affiliations:** 1 School of Engineering and Materials Science, Queen Mary University of London, London, E1 4NS UK; 2 University of Leicester, Leicester, LE1 7RH UK

**Keywords:** Applied mathematics, Computer science, Aerospace engineering

## Abstract

Multi-graphs where several edges connect a pair of nodes are an important modelling approach for many real-world optimisation problems. The multi-graph structure is often based on infrastructure and available connections between nodes. In this study, we conduct case studies for a special type of constrained routing and scheduling problems. Using the airport ground movement problem as an example, we analyse how the number of parallel edges and their costs in multi-graph structure influence the quality of obtained solutions found by the routing algorithm. The results show that the number of parallel edges not only affects the computational complexity but also the number of trade-off solutions and the quality of the found solutions. An indicator is further proposed which can estimate when the multi-graph would benefit from a higher number of parallel edges. Furthermore, we show that including edges with dominated costs in the multi-graph can also improve the results in the presence of time window constraints. The findings pave the way to an informed approach to multi-graph creation for similar problems based on multi-graphs.

## Introduction

Many optimisation problems in transportation, logistics or telecommunications can be formulated as search on a multi-graph. An example of problems include the vehicle routing problem, hazardous material transportation, multimodal shortest path problem and airport ground movement problem to name a few. The multiple parallel edges between pairs of nodes of the multi-graph offer a convenient way of modelling the real-world structure and inherent multi-objective nature of the problems including time, economic or environmental objectives. The parallel edges can represent routes with different costs in the multi-objective vehicle routing problem^1–3^ and hazardous material transportation^4^, different modes of transport^5,6^ in the multimodal shortest path problem and tour planning^7^, or different speed profiles in the trajectory based traffic management^8–10^. Furthermore, there are often various constraints which have to be satisfied by the solutions in order to be feasible, for example a delivery vehicle must visit customers in specified time windows.

So far, the research on the abovementioned optimisation problems focused mainly on the search algorithms for finding the best solutions using the multi-graph formulation of the problem. The structure of the multi-graph has been considered given and fixed, representing the real-world connections between the nodes. This is to some extent true for some problems such as the vehicle routing or multimodal shortest path problem where the multi-graph represents the underlying and existing infrastructure (roads, rail lines, etc.). However, even for these problems, infrastructure and schedule design is worth of investigation. For other problems such as the trajectory based traffic management, the multi-graph structure is mainly a result of the modelling approach, where the number of the parallel edges and their costs are design parameters. For example in^10^, there is an infinite number of speed profiles between two nodes (corresponding to different continuous speeds) and the multi-graph can include only some of them. As noted in the previous research^11^, the number of the parallel edges and their costs not only affect the computational times of the search algorithms but also the quality of the obtained solutions.

In this study, we further analyse the cases and conditions when the multi-graph structure has a direct consequence for the search algorithm. The case study of the airport ground movement problem is used to demonstrate how the decision on the number of the parallel edges and their costs affect the quality of the found solutions. The results in turn can inform the creation of the multi-graph not only for the airport ground movement problem but also other problems such as the above mentioned vehicle routing problem, hazardous material transportation, the multimodal shortest path problem and trajectory based traffic management. For example, the need to determine the number of multi-objective speed profiles for the trajectory based traffic management^8–10^ and the vehicle routing problem including energy-efficient driving^6,9^ plays a critical role in providing solutions, as it does in the airport ground movement.

**Figure 1:**
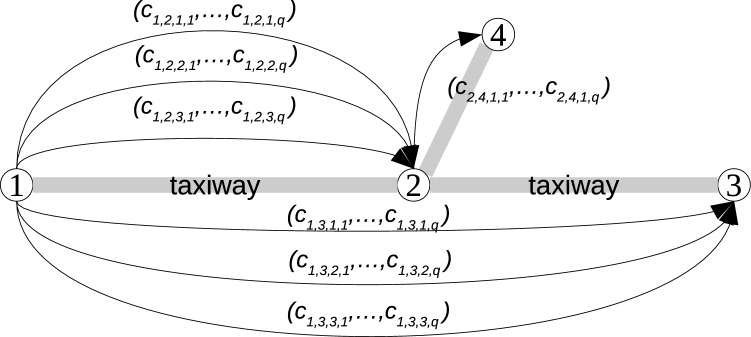
Each segment between nodes *n*, *m* has *u* speed profiles and a corresponding cost matrix $C_{n,m} \in \mathbb {R}^{u \times q}$ with size $u \times q$ associated with it, where *u* and *q* are the number of speed profiles and objectives, respectively. The segments are the basic unit in constructing a multi-graph.

The airport ground movement problem is a combined routing and scheduling problem which aims to find conflict-free routes and schedules for all aircraft taxiing between gates/stands and runway or vice versa with minimum taxi time and fuel consumption. The airport taxiway layout corresponds to a simple graph, which is expanded into a multi-graph by considering segments (i.e. a sequence of edges of the same type such as straight and turning) and their associated speed profiles as shown in Fig. 1.

The routing algorithm uses the multi-graph for the search and has to select: (1) which segments to include in the route between the start and end nodes; (2) which speed profiles to use for the selected segments. In order to prevent conflicts, each edge can be occupied only when it or a nearby edge is not traversed by another aircraft. For this purpose, aicraft must satisfy a time constraint called time window on each edge of its route. In this paper, we consider two objectives: $obj_1$ is the taxi time and $obj_2$ is the fuel consumption. A specialised routing algorithm AMOA*^11^ based on multi-objective A* algorithm is used in this paper. It should be noted, that any search algorithm, e.g. metaheuristic^12^, can be employed for this purpose.

The routing algorithm considers aircraft iteratively according to their start times. This first-come-first-served sequencing has an advantage to consider aircraft sequentially as they become ready to start taxiing. For each aircraft, a set of routes with nondominated costs are found by the algorithm using edges with feasible time windows. The edges of infeasible time windows are avoided by the routing algorithm, causing a detour. If no route is found due to the lack of available time windows, the start time of the aircraft is postponed by 60 s. This value is set approximately as airports usually operate (e.g. estimated time of departure) with a precision in minutes^11^. The start time is iteratively extended until time windows become eventually available. Once a set of routes is obtained, one route with the minimum cost is selected. The minimum cost is calculated by multiplying the values of $obj_1$ and $obj_2$ with corresponding unit costs $w^P=(0.469,0.71)$. The unit cost of 0.469 EUR/s for taxi time was calculated in^13^ and includes the cost for maintenance, fleet and crew. The unit cost for fuel consumed is set to 0.71 EUR/kg as in^13^. It should be noted, that $w^P$ is not utilised within AMOA*. $w^P$ used here replaces a decision maker who would in real operation select a route for each aicraft from a set of Pareto routes according to his/her preferences. After the route is selected, time windows are updated for edges belonging to its segments. To ensure a safe separation from other aircraft, also time windows of edges within a threshold distance of 60 m are blocked in addition to the edges of the selected route. The separation of 60 m corresponds to approximately 12 s difference between successive aircraft at taxiing speed 10 knots, similarly as in^13^.Table 1 summarises the definitions used in this paper.

**Table 1:** Notations used throughout the paper.

Variable	Description
$obj_1$, $obj_2$	The objectives, i.e. taxi time and fuel consumption
$t_1,t_2$	The fastest/slowest time at which the aircraft can arrive at the edge
$I_1,I_0$	The number of routes that can/cannot be potentially improved by using different speed profiles
$I_{eff}$	The ratio of improved routes to $I_1$
*cost*	The total cost

Parameter	Description
$G=(V,E)$	The directed graph of airport taxiways with nodes $n \in V$ and edges $e \in E$
$s_{n,m}=(e_{1},e_{2},\ldots )$	A segment which is a sequence of edges connecting two nodes *n*, *m*
*u*	The number of speed profiles
*q*	The number of objectives
$C_{n,m}$	The cost matrix of a segment between two nodes *n*, *m* with size $u \times q$
$c_{n,m,l,*}$	Cost vector, i.e. the *l*th speed profile for segment *n*, *m*
*M*	A large number
$w^P=(w^P_1,w^P_2)$	The vector of preferences (i.e. weights for objectives) representing unit costs

## Results

### Airport ground movement problem instances

**Figure 2:**
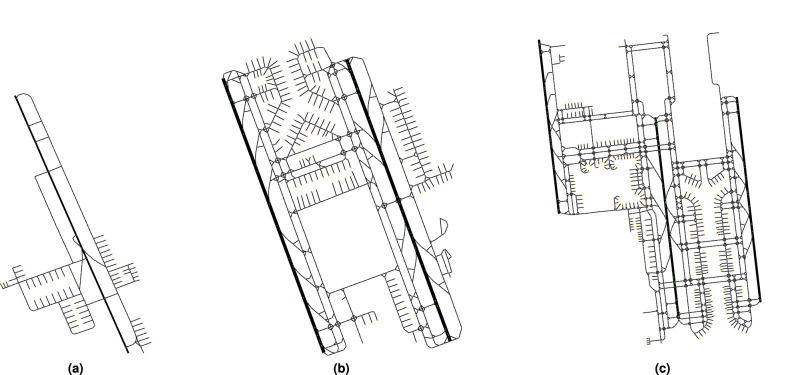
A directed graph representation of the airport surface for (**a**) Doha International Airport, (**b**) Hong Kong International Airport, (**c**) Beijing Capital International Airport.

In this paper, we use a set of real instances of arrival and departure flights from 3 airports: Doha International Airport (DOH), Hong Kong International Airport (HKG) and Beijing Capital International (PEK). The complexity of the taxiway layout ranges from simple (DOH), medium (HKG) to complex (PEK), as shown in Fig. 2. The graphs and flights are detailed in Tables 2 and 3 . The data specifies landing/pushback times, gates/runway exits and the weight category for each flight. The instances contain traffic data as follows. Instances marked *original* are from our previous work^11^. The instance *ins*1 is an artificial instance with introduced aircraft conflicts. *ins*2 is similar to *ins*1 but with conflicting aircraft isolated such that the aircraft later in the sequence are not affected by routes of previous aircraft. Finally, instances with increased traffic (0–100 %) are used. The instances with introduced aircraft conflicts and higher traffic are expected to have less available time windows which may benefit from a multi-graph structure with higher *u*. The process of creating the airport layout and capturing the flight data is detailed in the “Methods” section.

**Table 2:** Airport layout instances.

	Nodes	Edges	Gates	Runway exits
DOH	434	436	55	14
HKG	1309	1491	160	38
PEK	3194	3928	286	53

**Table 3:** Aircraft traffic instances.

Instance	Aircraft	Instance	Aircraft	Instance	Aircraft
doh *original*	180	hkg *original*	506	pek *original*	349
doh ins1	62	hkg ins1	60	pek ins1	52
doh ins2	62	hkg ins2	60	pek ins2	52
doh0	52	hkg0	60	pek0	91
doh25	65	hkg25	75	pek25	114
doh50	78	hkg50	90	pek50	137
doh75	91	hkg75	105	pek75	159
doh100	104	hkg100	120	pek100	182

### Time complexity and Pareto front

**Figure 3:**
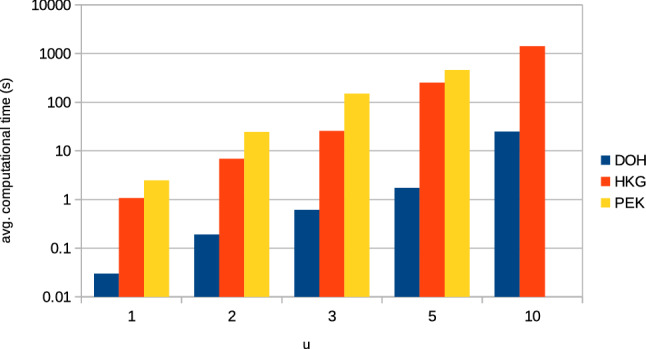
Computational times for a single aircraft in seconds for varying *u*. Note, that for PEK and $u=10$ the experiments could not be completed within 10 days limit.

**Figure 4:**
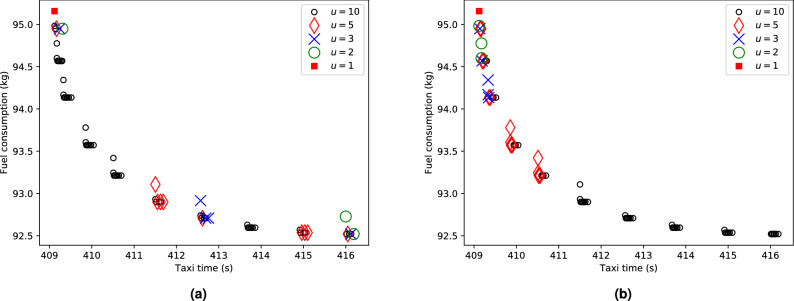
Pareto front for a single aircraft: (**a**) solutions obtained by AMOA* with multi-graph reduction based on evenly distributed solutions and increasing *u*, (**b**) solutions obtained by AMOA* with preferences with $w^p=(M,0)$, where *M* is a large number and increasing *u*.

Besides the number of nodes in the multi-graph (depending on the airport), the number of parallel edges corresponding to the speed profiles (the size of $C_{n,m}$) significantly affects the size of the search space. As mentioned above, there is a large number of speed profiles (in this study, we used 20) available for each segment. As a result, the complexity rises exponentially as shown in Fig. 3 and experiments with more than 5 speed profiles for each segment could not be finished in reasonable time for all airport instances. To address this problem, two different speed profile selection approaches are used to reduce the multi-graph. Figure 4a,b illustrate the difference of speed profile selection for increasing number of *u* for a route of a single aircraft. The number of found Pareto optimal solutions gradually grows with the larger value of *u*. With evenly distributed speed profiles, the found solutions gradually cover the whole Pareto front with a larger *u*. The same is true for the preference-based selection of speed profiles, where the solutions concentrate at the preferred region with a smaller *u* and gradually spread to cover the whole Pareto front with a larger *u*. Both speed profile selection approaches are described in detail in the “Methods” section.

### Effect of time windows

So far we have seen that *u* and the selection of speed profiles affected the number of obtained solutions and the associated time complexity. However, the above mentioned experiments in Figs. 3 and 4 were conducted for an unconstrained case as only one aircraft is considered. In the case of multiple aircraft, a solution for one aircraft has an effect on the subsequent aircraft due to constraints as represented by time windows. In such a case, the choice of larger values of *u* enables the algorithm to consider alternative speed profiles which may comply with the time windows, leading to better solutions.

**Table 4:** Results for experiments with different values of *u*.

Instance	$u=3$		$u=5$		$I_1$	$I_0$	$I_{eff}$
	Cost	Improved	Cost	Improved			
doh original	− 0.67%	2	− 0.46%	2	7	1	28.57%
doh ins1	1.21%	2	1.23%	1	8	0	12.50%
doh ins2	0.51%	1	0.98%	1	5	0	20.00%
doh0	0.11%	2	0.31%	2	4	0	50.00%
doh25	0.93%	4	1.17%	4	8	0	50.00%
doh50	− 0.19%	3	− 0.14%	3	7	1	42.86%
doh75	0.06%	11	0.74%	9	11	5	81.82%
doh100	1.82%	22	4.10%	26	30	5	86.67%
hkg original	− 0.35%	14	− 0.35%	14	58	11	24.14%
hkg ins1	2.37%	14	2.83%	10	30	0	33.33%
hkg ins2	1.61%	13	2.33%	8	28	1	28.57%
hkg0	0.59%	6	0.66%	5	12	0	41.67%
hkg25	0.16%	5	0.31%	5	16	0	31.25%
hkg50	0.20%	11	0.47%	14	28	1	50.00%
hkg75	0.02%	16	1.16%	19	42	3	45.24%
hkg100	3.03%	26	4.12%	32	55	2	58.18%
pek original	− 0.07%	7	0.17%	8	36	6	22.22%
pek ins1	0.19%	6	1.06%	7	17	3	41.18%
pek ins2	1.13%	6	0.83%	5	17	2	29.41%
pek0	− 0.54%	1	− 0.27%	1	7	1	14.29%
pek25	− 0.37%	4	− 0.3%	4	16	2	25.00%
pek50	0.92%	11	1.12%	10	25	5	40.00%
pek75	1.50%	20	1.55%	17	32	3	53.13%
pek100	0.25%	16	1.28%	20	63	10	31.75%

In order to analyse the effect of *u* on the quality of the obtained results, a baseline with $u=1$ is established. The multi-graph is reduced to a single-graph with a single speed profile for each segment. The preference $w^P=(0.469,0.71)$ is used to select the speed profile and for reserving a route for each aircraft. Further experiments were conducted with $u=3$ and $u=5$, where the speed profiles for each segment were selected evenly. In order to facilitate the comparison, Table 4 shows savings of total cost (calculated as $obj_1 \cdot 0.469+ obj_2 \cdot 0.71$) obtained with $u=3$ and $u=5$ compared to the baseline case. The values of objectives are in “Appendix” section. Positive values in column *cost* refer to the cost saving. The column marked as ‘improved’ refers to the number of aircraft better in both objectives with respect to $u=1$. In order to estimate the number of aircraft that can be improved, indicators $I_1$ and $I_0$ are proposed with more details in “Methods” section. $I_1$ is the number of routes which can be potentially improved using alternative speed profiles. $I_0$ is the number of routes which cannot be improved even by using alternative speed profiles. Negative values of *cost* refer to higher costs than those of the baseline case with $u=1$. As more speed profiles are included in the multi-graph with $u>1$ than the baseline case, finding worse routes should not be possible. Worse routes are caused by sequential routing of aircraft. In individual cases, the routing algorithm searching the multi-graph with larger *u* can always find a route with better costs. However, this can have detrimental effect on the availability of edges for the subsequent aircraft, resulting in a detour and higher objective values.

To gain more insight, firstly, we analyse the instances marked *original*. The higher values of *u* resulted in mostly negative savings $<1\%$ which are caused by sequential routing of aircraft as explained above. The lack of improvement can be also explained by fewer conflicts among aircraft leading to a fewer number of edges with infeasible time windows. The lower number of conflicts is evident from the sum of columns $I_1$ and $I_0$ which indicate the number of potential conflicts on the unimpeded route. The conflicted aircraft range from 4% to 14% in instances marked *original*. As a result, speed profiles in the multi-graph with $u=1$ can comfortably satisfy time window constraints in most cases.

Secondly, we analyse the instances marked *ins*1 and *ins*2. These instances denote artificial scenarios created as detailed in “Methods” section which introduce conflicts among aircraft on purpose. The higher proportion of conflicts in the instances resulted in larger relative savings up to around 3% with higher values of *u*, particularly for the HKG airport. The savings with $u=5$ are higher than for $u=3$. In most cases, *ins*2 instances obtained less savings compared to *ins*1. Instances in *ins*2 are similar to *ins*1 but with less conflicts caused by sequential routing of aircraft. The sequential routing can lead to both better or worse routes for the subsequent aircraft. The difference in results for *ins*1 and *ins*2 reflects the effect of sequential routing. As can be seen, even without this effect, results for *ins*2 show that the higher proportion of conflicts compared to *original* instances, resulting in larger savings.

Lastly, artificial instances with increased traffic are investigated. These instances are marked 0–100 denoting the % increase in traffic over the *original* levels at the rush hour. When the traffic is high, several aircraft routes are likely to be in conflict with each other. The results show mostly positive values of savings which are increasing with higher traffic. As with previous experiments, the savings with $u=5$ are higher than for $u=3$. The trend in improvement with higher levels of traffic is clearly documented in the increasing number of routes denoted as ‘improved’. As the traffic increased, the number of conflicts and potential routes which can be improved with more speed profiles are increased too. For DOH and PEK airports, the % savings are both positive and negative. The DOH instances have less conflicts (as indicated in the values of $I_1$ and $I_0$) compared to other airports. Also, the airport layout is simple and the routing algorithm has less options to take a detour. Instead, the routing algorithm with $u=1$ delays the start time of an aircraft where the extra waiting time does not constitute additional fuel costs as it happens at the start/end of the route. For departures this is achieved at the gate with engines turned off. For arrivals, it is assumed that postponing can be achieved before landing via air traffic control procedures and therefore not affecting fuel costs at ground. With a higher *u*, the route can be found without postponing but with higher costs than $u=1$ and extension. For PEK airport, the magnitude of savings and improved routes are both lower than for HKG. This can be explained by a more complicated layout of PEK than HKG. As a result, the opportunity of taking a detour is more frequent and less penalising (shorter detours possible) than at HKG. This causes less savings for the routes without detours found with higher *u*.

The indicated number of routes which can be improved in column $I_1$ is higher than the number of routes which are actually improved. The ratio $I_{eff}$ which is the number of ’improved’ routes to $I_1$ when $u=5$ ranges from around 13% to 87%. The ratio of $I_1$ to the total number of aircraft in the instance ranges 4–50%. This means, that if the proposed indicator is used only 4–50% of original aircraft have an opportunity for improvement with higher values of *u*.

### Dominated speed profiles

**Figure 5:**
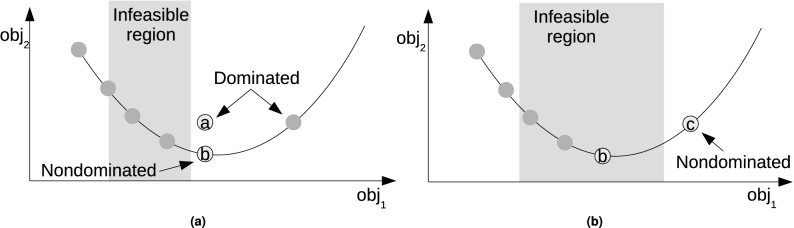
Pareto front for a single segment where the dots on the curve represent the evenly distributed speed profiles stored in the database. (**a**) Infeasible region covers the middle part of the Pareto front. (**b**) Infeasible region extends beyond the last nondominated solution (solution *b*) on the Pareto front.

The experiments presented in Table 4 used the multi-graph with nondominated speed profiles. This makes sense if there are no time window constraints. Figure 5a shows an objective space for a segment. If we assume that the Pareto front for speed profiles is continuous then any solution can be generated on the indicated curve. The assumption comes from the fact that the variables for the speed profile generation are continuous^14^. The curve has usually a parabolic shape and the left part constitutes the Pareto front. The right part has an upward trend, as speed profiles with very long taxi time after some threshold consume more fuel than faster speed profiles. For any feasible dominated solution (solution *a*), there is a corresponding nondominated solution (solution *b*) with the same taxi time and better fuel consumption. Therefore, to reach any solution on the feasible part of the Pareto front, only nondominated solutions located on this front have to be included in the multi-graph. However, when the infeasible region covers a larger portion of the Pareto front and extends beyond the last nondominated solution (solution *b*) as in Fig. 5b, the previously dominated speed profile (solution *c*) becomes nondominated. Therefore, to reach this new part of the Pareto front, the previously dominated speed profile needs to be included in the multi-graph.

**Figure 6:**
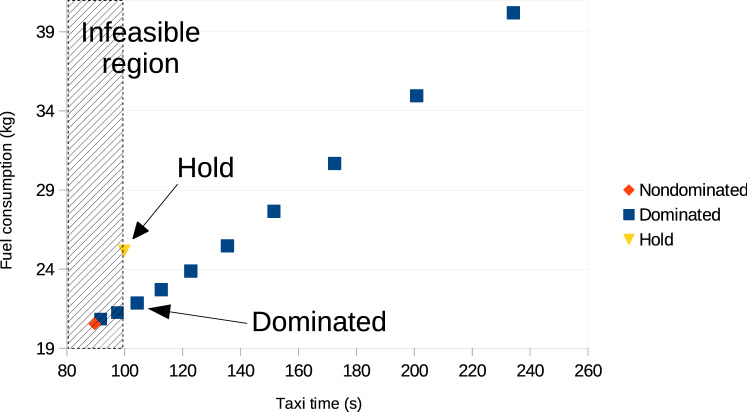
For resolving the infeasible nondominated speed profile, holding can be applied (at additional cost) or another feasible speed profile with the least cost from the dominated speed profiles can be selected.

The presence of time window constraints affects the search in AMOA*. If some edge does not have any time window available for the current speed profile, then holding can be applied (at additional cost) or another speed profile can be selected if available. In the following, we conducted experiments on a multi-graph with $u=1$, $u=3$ and $u=5$ with holding where the aircraft is held at the end of the previous segment with engines running at idle until the time window becomes feasible. Also, in another set of experiments, instead of holding, the routing algorithm checks the database of dominated speed profiles and selects one with the least cost which is feasible. Both approaches are illustrated in Fig. 6. It should be noted that in both cases we are effectively adding a dominated speed profile into the multi-graph.

Table 5 details cost savings obtained with applied holding and dominated solutions. The column headings (e.g. cost($u=3$)) denote the baseline case without holding against which the results are compared with. Columns marked cost($u=1$) show savings compared with the baseline case $u=1$ without holding. The values reflect cumulative savings from both increased *u* and using holding (dominated solutions). Columns marked cost($u=3$) and cost($u=5$) show savings compared to the baseline case with the same *u* and thus reflect the effect of holding and dominated solutions. Savings are positive for all instances and the number of improved routes is relatively high. In all cases, a dominated speed profile approach achieved better results than holding. This is expected, as a dominated speed profile avoids a fuel penalty for accelerating from rest as in holding. On the other hand, holding can have better cost savings when the dominated speed profile with comparable taxi time is missing due to the limited number of dominated speed profiles stored in the database. The feasible dominated speed profile may have higher costs than holding in such a case. The positive savings are particularly high for *ins* and higher traffic instances with up to 8.84% for DOH airport and $u=5$. Values in columns cost($u=3$) and cost($u=5$) show high savings and document a high potential for improvement by using holding and dominated speed profiles.

**Table 5:** Results for experiments with dominated speed profiles.

Instance	$u=1$		$u=3$			$u=5$		
	Cost($u=1$)	Improved	Cost($u=3$)	Cost($u=1$)	Improved	Cost($u=5$)	Cost($u=1$)	Improved
**Holding**								
doh original	1.91%	5	1.35%	0.69%	2	1.62%	1.17%	3
doh ins1	6.75%	5	5.66%	6.80%	4	6.15%	7.30%	5
doh ins2	5.28%	2	4.63%	5.12%	2	4.74%	5.68%	2
doh0	1.71%	3	1.05%	1.16%	0	1.38%	1.68%	1
doh25	2.02%	5	0.71%	1.64%	0	0.92%	2.08%	1
doh50	1.59%	5	0.76%	0.57%	2	1.42%	1.28%	2
doh75	4.56%	9	3.41%	3.46%	7	3.15%	3.87%	12
doh100	5.32%	25	6.15%	7.86%	21	4.56%	8.47%	18
hkg original	0.78%	21	0.77%	0.43%	18	0.82%	0.47%	26
hkg ins1	6.94%	18	4.25%	6.52%	15	3.76%	6.48%	10
hkg ins2	6.11%	18	3.95%	5.50%	13	3.48%	5.73%	9
hkg0	1.35%	9	0.79%	1.38%	5	0.65%	1.30%	4
hkg25	0.88%	8	0.74%	0.90%	6	0.59%	0.89%	5
hkg50	2.09%	18	1.93%	2.13%	16	1.73%	2.20%	14
hkg75	2.73%	28	3.97%	3.99%	25	2.85%	3.98%	19
hkg100	5.24%	29	2.40%	5.36%	22	1.37%	5.44%	14
pek original	0.80%	11	0.64%	0.58%	8	0.65%	0.81%	7
pek ins1	1.78%	6	1.79%	1.98%	3	0.96%	2.01%	3
pek ins2	2.36%	8	1.66%	2.77%	3	1.72%	2.54%	5
pek0	0.66%	0	0.87%	0.34%	3	0.83%	0.56%	0
pek25	1.88%	6	1.97%	1.61%	8	2.16%	1.87%	6
pek50	3.37%	10	2.34%	3.23%	7	2.43%	3.52%	6
pek75	3.48%	15	1.94%	3.41%	7	2.12%	3.64%	4
pek100	2.58%	21	2.82%	3.07%	14	2.20%	3.45%	17
**Dominated**								
doh original	2.06%	6	1.45%	0.79%	4	1.75%	1.29%	5
doh ins1	7.85%	12	5.73%	6.86%	5	6.44%	7.59%	8
doh ins2	6.42%	9	4.70%	5.19%	3	5.04%	5.97%	5
doh0	2.06%	4	1.30%	1.41%	1	1.68%	1.98%	2
doh25	2.40%	7	0.93%	1.85%	2	1.13%	2.28%	2
doh50	2.14%	6	1.03%	0.85%	3	1.69%	1.55%	2
doh75	5.38%	19	3.82%	3.88%	13	3.58%	4.29%	17
doh100	5.73%	30	6.44%	8.15%	22	4.94%	8.84%	21
hkg original	0.84%	20	0.84%	0.49%	19	0.90%	0.55%	27
hkg ins1	7.26%	18	4.72%	6.98%	17	4.11%	6.83%	13
hkg ins2	6.31%	18	4.25%	5.79%	15	3.68%	5.93%	10
hkg0	1.36%	8	0.85%	1.43%	5	0.72%	1.37%	4
hkg25	1.00%	7	0.87%	1.03%	6	0.72%	1.02%	5
hkg50	2.16%	17	2.05%	2.25%	15	1.89%	2.35%	13
hkg75	3.15%	28	4.17%	4.18%	27	3.12%	4.24%	21
hkg100	5.72%	32	2.71%	5.66%	22	1.78%	5.83%	16
pek original	0.92%	12	0.71%	0.64%	10	0.70%	0.86%	10
pek ins1	2.24%	8	1.98%	2.17%	6	1.05%	2.09%	3
pek ins2	2.74%	9	1.82%	2.92%	5	1.98%	2.79%	7
pek0	0.84%	2	0.97%	0.44%	4	0.92%	0.65%	3
pek25	2.21%	10	2.20%	1.84%	12	2.33%	2.04%	12
pek50	3.68%	13	2.51%	3.40%	13	2.59%	3.69%	12
pek75	3.79%	18	2.17%	3.64%	13	2.37%	3.88%	12
pek100	3.04%	25	3.14%	3.38%	28	2.50%	3.75%	29

## Discussion

In this study, we analysed cases and conditions when the multi-graph structure directly influences the quality of the obtained solutions found by a routing algorithm. The airport ground movement problem was employed as a case study. The experiments were carried out with the different number of the parallel edges and costs.

The higher number of parallel edges resulted in higher number of Pareto optimal solutions found and time complexity of the search. Different approaches for speed profile selection could control which parts of the Pareto front are covered by the solutions.

For the *original* instances, the increased *u* resulted in negative or only small improvements $<1\%$. However, artificial instances with higher number of conflicts showed higher savings up to 4.12% in some cases. With higher traffic, the savings increased too. This result suggests that when traffic levels are low the multi-graph with $u=1$ can find good quality solutions in most cases. As search with $u=1$ is fast compared to a case with higher *u*, using $u=1$ is preferential. Therefore, the routing algorithm can be adopted for a multi-graph with $u=1$. In this case, the multi-graph can be constructed using preferences $w_p$ determined by the decision maker, or $w_p$ can be iteratively changed to cover different parts of the Pareto front. For the higher traffic levels, $u=3$ can improve the results and $u=5$ even more.

An indicator proposed to estimate the number of routes which can be potentially improved by using alternative speed profiles could be successfully used to indicate in which case a higher value of *u* is beneficial. Therefore, the routing algorithm should use a higher values of *u* only for those instances (i.e. aircraft), while for others, $u=1$ is sufficient. The ratio of $I_1$ to the total number of aircraft in the instance ranged 4–50%. If $u>1$ is used only for those aircraft indicated in $I_1$, 50–96% aircraft can use $u=1$ and save computational time.

Also, the results highlighted the differences in savings between different airports, pointing to the importance of the airport layout. On the other hand, as savings with higher *u* demonstrate, using more complex modelling techniques can bring benefits in high traffic scenarios and potentially offset costly investments in new taxiways.

The experiments with dominated speed profiles showed the importance of considering including dominated parallel edges in a multi-graph in the presence of time window constraints. With dominated speed profiles included, the routing algorithm could find routes up to 7.85% better compared to the multi-graph with the same *u* without dominated speed profiles.

In future work, a similar case study could shed light on the creation of the multi-graph for other problems based on multi-graphs such as the vehicle routing problem, hazardous material transportation, the multimodal shortest path problem and trajectory based traffic management. Also, the routing algorithm could actively use the indicator or some similar measure to create multi-graphs with a structure which is fast for search but guarantees high quality solutions. The results in this study also highlighted the issue of sequential routing when the route of one aircraft can affect the subsequent aircraft. One way of addressing this problem is to search for a better sequence of aircraft. However, even more promissing global approach would be considering multiple aircraft simultaneously such that the multi-graph structure could be utilised to avoid conflicts not only with subsequent aircraft but all aircraft considered.

## Methods

### Multi-graph

The airport layout is represented as a directed graph $G=(V,E)$. Nodes $n \in V$ represent gates, stands, taxiway intersections, intermediate points and runway exits. Edges $e \in E$ represent taxiways between two nodes. A sequence of edges of a similar type between two nodes *n*, *m* is defined as a segment $s_{n,m}=(e_{1},e_{2},\ldots ,e_{h})$. The segments are of two types, straight and turning. If an edge and its predecessor edge (in the direction of a taxiing aircraft) have an angle $\ge 30$ degrees^15,16^, then it will belong to a turning segment. Otherwise, it is part of a straight segment. Consecutive edges of a similar type (straight, turning) are grouped together. For a segment between two nodes *n*, *m*, $(c_{n,m,l,1},c_{n,m,l,2},\ldots ,c_{n,m,l,q}) \in C_{n,m}$ is a cost vector with *q* objectives which corresponds to the *l*th speed profile for that segment. Speed profiles for a single segment are continuous functions of time^14^. In this study, a piece-wise linear function with four phases^14^ including acceleration, constant speed, deceleration and rapid deceleration is adopted. The duration of each phase and the associated thrust levels determine the taxi time and fuel consumption of the speed profile. Evenly distributed speed profiles according to the two objectives from Pareto solutions for all segments were adopted in our previous study^17^. The speed profiles include different weight categories of aircraft for up to 20 speed profiles per segment. The multi-graph is constructed using these speed profiles.

As multi-graphs with a large number of speed profiles per segment can be computationally prohibitive, two different approaches are proposed to reduce the multi-graph: (1) From the Pareto front of speed profiles for a segment, *u* evenly distributed solutions can be considered. In this study, rows in $C_{n,m}$ were ordered according to the first objective and *u* solutions were selected with even distance from each other according to the first objective. (2) If preferences for the search are known beforehand, the first *u* speed profiles ranked according to that preference can be considered. In this study, we take the scalar product of $obj_1$ and $obj_2$ in each row in $C_{n,m}$ and unit costs $w^P$ as the preferences. These aggregated costs then give the ranking of the rows in $C_{n,m}$ and lower costs are preferred. As an example, consider a Pareto front of speed profiles with two objectives: {(1,6),(2,4),(4,3),(5,2),(7,1)} where we want to select $u=3$ solutions. In the case of evenly distributed solutions, we select the extreme solutions (1,6) and (7,1) and the middle solution (4,3) which has the same distance from the extreme solutions w.r.t. the first objective. This way, the Pareto front is evenly covered with our selection. In the case of known preferences, e.g. $w^p=(M,0)$ where *M* is a large number, we select (1,6) with the lowest cost and two solutions (2,4) and (4,3) with the 2nd and 3rd lowest cost, respectively.

### Airport layout

The taxiway layout was processed^18^ from OpenStreetMap (www.openstreetmap.org). All edges were set as bi-directional. The flights for DOH and HKG instances were captured with the specialised tools^18^ from freely-available data on FlightRadar24.com. The data specifies landing/pushback times, gates/runway exits and weight category for each flight.

### Aircraft instances

Instances marked *original* denote problems from^11^. These contain flights from 16.3.2014 (17:00–23:00) for DOH, 17.1.2017 (0:00–24:00) for HKG and 9.7.2014 (9:00–14:00) for PEK.

The instances marked *ins*1 and *ins*2 are artificial instances. From the *original* instances, pairs of aircraft with overlapping unimpeded routes were selected and their start time changed such that they arrived to where their routes intersected at the same time . When the traffic is high, several aircraft routes can be in conflict with each other. In order to eliminate such interference, pairs of aircraft from *ins*2 are separated from other pairs by a large time interval.

The instances marked 0–100 were generated as follows. From the original instances, 1 hr of traffic during rush hour was selected, marked 0 (e.g. doh0). Then, artificial instances were generated with traffic levels increased by 25, 50, 75 and 100% by randomly adding additional aircraft. It should be noted that some instances would be unrealistic due to the fact that the runway is usually the main bottleneck at airports. With a theoretical maximum capacity of 60 aircraft per hour for a single runway, some instances for HKG (2 runways in total) and DOH (1 runway) could exceed this capacity.

### Improvement indicator

An indicator is proposed in this paper to indicate the number of routes that could be improved by using alternative speed profiles. The indicator is outlined in Algorithm 1. For an aircraft, the routing algorithm is run in Line 1 to find and select a route (using the same $w^P=(0.469,0.71)$ as above) without considering any time windows (the unimpeded route). This step can be carried out offline before the search. Then, during the search the routing algorithm is run again in Line 2 considering time windows. The resulting route is compared with the unimpeded route in Line 3. If the two routes are identical, then the route is already the best route and cannot be improved by using different speed profiles. Otherwise, the difference is caused by a conflict with another aircraft and the corresponding time window causing the conflict is found. Firstly, the first differing edge (in the direction from the start to destination node) is identified in Line 7. Then, the fastest speed profiles are applied in Line 8 to calculate the fastest time $t_1$ at which the aircraft can arrive at the identified edge. Furthermore, $t_2$ is computed as the time of applying the slowest speed possible (5.14 m/s, 10 knots) in Line 9. The interval between $t_1$ and $t_2$ corresponds to times at which the aircraft can arrive at the edge using different speed profiles on the previous segments. If this interval in Line 13 is wide enough to traverse the edge using minimum traversing time (the time using the fastest speed profile) and not in conflict with the time window, then the route is included in $I_1$ in Line 14. The route counted in $I_1$ can be potentially improved by avoiding the conflicting time window using different speed profiles. Otherwise, the route is included in $I_0$ in Line 16 because no matter which speed profile is used, the arrival and departure times from the edge will conflict with the time window. $I_0$ is therefore the number of routes which cannot be improved even by using alternative speed profiles. It should be noted, that $I_1$ only indicates a potential improvement and serves as a metric corresponding to the upper bound on how much the results can be improved using $u>1$ speed profiles. The alternative speed profile can be more costly than the detour and also any time window conflicts on subsequent edges are not tested. $I_{eff}$ is the ratio of actually improved routes to $I_1$ using $u>1$ speed profiles. The actually improved routes used alternative speed profiles to avoid the conflicting time window and the subsequent detour which resulted in better objectives.
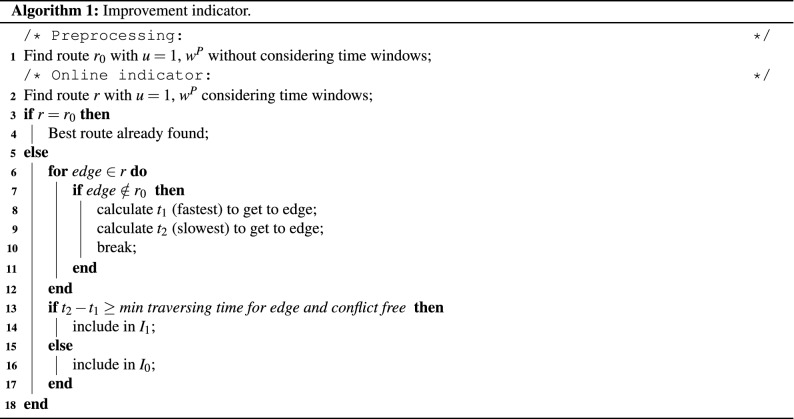


It should be noted, that the proposed indicator does not compromise the search. If there are no conflicting time windows, the selected route from the Pareto front found by the routing algorithm with $u=1$ and $u>1$ are the same due to the minimum cost being the same. This condition is tested in Line 3. Otherwise, the indicator algorithm tests if it is feasible to prevent the conflicting time window. In the case of $I_1$, the conflict can be prevented with $u>1$ and full search should be conducted. In the case of $I_0$, the conflict is inevitable and therefore search with $u>1$ cannot find a better route.

## Data Availability

The airport layout data sets and anonymised aircraft traffic instances are available here: https://github.com/mweiszer/amoa_reports/blob/main/data.zip.

## Appendix

See Tables 6 and 7.

**Table 6:** Full results for experiments with different values of *u.*

Instance	$u=1$		$u=3$		$u=5$	
	$obj_1$	$obj_2$	$obj_1$	$obj_2$	$obj_1$	$obj_2$
doh original	34,402	14,431	34,581	14,561	34,611	14,464
doh ins1	13,999	4846	13,698	4875	13,772	4823
doh ins2	13,356	4776	13,188	4818	13,190	4752
doh0	12,608	5255	12,606	5241	12,576	5234
doh25	16,637	7098	16,479	7034	16,441	7016
doh50	20,986	8597	20,999	8631	21,099	8554
doh75	26,984	10,429	26,811	10,527	26,404	10,604
doh100	32,830	12,748	31,599	12,933	31,010	12,538
hkg original	134,001	64,120	134,393	64,389	134,393	64,389
hkg ins1	14,293	6933	13,857	6833	13,845	6765
hkg ins2	13,743	6921	13,450	6857	13,398	6776
hkg0	16,971	8178	16,899	8111	16,986	8041
hkg25	21,301	10,174	21,268	10,156	21,364	10,058
hkg50	26,595	12,743	26,557	12,707	26,569	12,618
hkg75	33,060	15,420	33,104	15,385	32,749	15,193
hkg100	37,562	17,532	36,318	17,069	36,166	16,708
pek original	91,066	28,158	91,055	28,225	91,208	27,918
pek ins1	15,302	4445	15,385	4362	15,307	4288
pek ins2	14,944	4393	14,825	4311	14,899	4304
pek0	22,521	7205	22,588	7280	22,653	7178
pek25	31,314	9719	31,360	9800	31,500	9687
pek50	38,981	11,636	38,566	11,567	38,665	11,425
pek75	46,338	13,901	45,602	13,719	45,836	13,544
pek100	56,308	16,643	56,188	16,589	55,807	16,284

**Table 7:** Full results for experiments with dominated speed profiles.

Instance	Holding						Dominated					
	$u=1$		$u=3$		$u=5$		$u=1$		$u=3$		$u=5$	
	$obj_1$	$obj_2$	$obj_1$	$obj_2$	$obj_1$	$obj_2$	$obj_1$	$obj_2$	$obj_1$	$obj_2$	$obj_1$	$obj_2$
doh original	33,392	14,387	33,782	14,584	33,710	14,453	33,409	14,322	33,791	14,541	33,722	14,400
doh ins1	12,249	5051	12,208	5071	12,178	5020	12,260	4889	12,208	5061	12,181	4978
doh ins2	11,988	4961	11,977	4991	11,933	4944	11,998	4800	11,977	4981	11,936	4902
doh0	12,184	5303	12,262	5326	12,230	5276	12,195	5248	12,271	5287	12,239	5230
doh25	16,083	7098	16,154	7121	16,141	7050	16,115	7008	16,179	7066	16,169	6994
doh50	20,077	8841	20,383	8867	20,272	8782	20,136	8678	20,443	8765	20,332	8680
doh75	24,698	10,652	25,086	10,704	25,021	10,633	24,801	10,352	25,178	10,526	25,155	10,425
doh100	29,754	12,947	28,922	12,622	28,829	12,473	29,855	12,742	29,022	12,458	28,929	12,281
hkg original	132,385	64,001	132,802	64,255	133,163	63,950	132,493	63,839	132,873	64,114	133,259	63,765
hkg ins1	12,983	6662	13,016	6709	13,104	6657	13,003	6596	13,033	6623	13,126	6586
hkg ins2	12,822	6552	12,876	6614	12,895	6565	12,840	6509	12,885	6561	12,915	6519
hkg0	16,664	8120	16,667	8112	16,759	8066	16,691	8100	16,672	8097	16,761	8051
hkg25	20,946	10,194	20,948	10,188	21,053	10,121	20,973	10,149	20,952	10,155	21,055	10,089
hkg50	25,594	12,770	25,588	127,62	25,686	12678	25,651	12,710	25,619	12,706	25,710	12,617
hkg75	31,522	15,417	31,126	15,211	31,269	15,120	31,652	15,177	31,203	15,089	31,353	14,968
hkg100	34,722	17,189	34,827	17,069	34,917	16,975	34,807	16,929	34,893	16,898	34,993	16,761
pek original	90,003	28,152	90,150	28,254	90,298	27,950	90,026	28,031	90,194	28,165	90,341	27,876
pek ins1	14,842	4490	14,844	4459	14,875	4435	14,850	4418	14,844	4432	14,881	4419
pek ins2	14,426	4398	14,394	4361	14,444	4361	14,437	4338	14,407	4331	14,473	4306
pek0	22,243	7244	22,294	7280	22,355	7191	22,249	7199	22,324	7238	22,364	7166
pek25	30,248	9851	30,311	9892	30,383	9766	30,287	9725	30,358	9791	30411	9696
pek50	36,977	11,701	37,044	11,707	37,075	11,578	37,012	11,559	37,098	11,608	37,098	11,502
pek75	43,764	14,051	43,819	14,048	43,881	13,906	43,835	13,867	43,897	13,893	43,963	13,743
pek100	53,645	17,011	53,383	16,925	53,359	16,734	53,706	16,726	53,434	16,720	53,419	16,531
